# Baltimore’s Water Festival

**Published:** 1881-09

**Authors:** 


THE OCTOBER FESTIVAL, in honor of the completion of the waterworks, and the introduction of the water from the Gunpowder river into the City of Baltimore, promises to be a pageant of unusual grandeur and magnificence, probably unsurpassed in the annals of this country, save by that which created such a sensation in the city last autumn on the completion of its sesqui-centennial year.

The proposed demonstration is a fitting one, and will find its place in a high niche, among the many and grand achievements which have been already accomplished by the citizens of Baltimore. The magnitude of the work accomplished, in bringing the water of the Gunpowder river into Baltimore, and in such quantity as to meet the wants of its citizens even when the number shall have extended into the millions, stands without a rival or peer within the historic period! We shall recur to the subject again.

CHICAGO, 1881.

Dr. B. M. WILKERSON,

Dear sir :

Inclosed please find a bill just passed our Legislature. We have been working twelve years to get it through, but have just succeeded.

Yours, fraternally,

TRUMAN W. BROPHY.

We congratulate the dentists of Illinois on the success which has crowned their efforts in procuring the passage of such a law, which ought to place their calling among the specialties of medicine. Slowly, but, we trust, surely, dentistry is rising from a mechanic art, to the dignity of a department of medical science.

